# A strategy to identify housekeeping genes suitable for analysis in breast cancer diseases

**DOI:** 10.1186/s12864-016-2946-1

**Published:** 2016-08-15

**Authors:** Tatiana M. Tilli, Cláudio da Silva Castro, Jack A. Tuszynski, Nicolas Carels

**Affiliations:** 1 Laboratório de Modelagem de Sistemas Biológicos, National Institute of Science and Technology for Innovation in Neglected Diseases (INCT/IDN, CNPq), Centro de Desenvolvimento Tecnológico em Saúde, Fundação Oswaldo Cruz, Rio de Janeiro, Brazil; 2 Tecnologia da Informação, Centro de Desenvolvimento Tecnológico em Saúde, Fundação Oswaldo Cruz, Rio de Janeiro, Brazil; 3 Department of Oncology, Faculty of Medicine & Dentistry, University of Alberta, Edmonton, AB T6G 1Z2 Canada; 4 Department of Physics, University of Alberta, Edmonton, AB T6G 2E1 Canada

## Abstract

**Background:**

The selection of suitable internal control genes is crucial for proper interpretation of real-time PCR data. Here we outline a strategy to identify housekeeping genes that could serve as suitable internal control for comparative analyses of gene expression data in breast cancer cell lines and tissues obtained by high throughput sequencing and quantitative real-time PCR (qRT-PCR).

**Methods:**

The strategy proposed includes the large-scale screening of potential candidate reference genes from RNA-seq data as well as their validation by qRT-PCR, and careful examination of reference data from the International Cancer Genome Consortium, The Cancer Genome Atlas and Gene Expression Omnibus repositories.

**Results:**

The identified set of reference genes, also called novel housekeeping genes that includes CCSER2, SYMPK, ANKRD17 and PUM1, proved to be less variable and thus potentially more accurate for research and clinical analyses of breast cell lines and tissue samples compared to the traditional housekeeping genes used to this end.

**Discussion:**

These results highlight the importance of a massive evaluation of housekeeping genes for their relevance as internal control for optimized intra- and inter-assay comparison of gene expression.

**Conclusion:**

We developed a strategy to identify and evaluate the significance of housekeeping genes as internal control for the intra- and inter-assay comparison of gene expression in breast cancer that could be applied to other tumor types and diseases.

**Electronic supplementary material:**

The online version of this article (doi:10.1186/s12864-016-2946-1) contains supplementary material, which is available to authorized users.

## Background

As is well characterized at the cellular level, one of the main features of cancer intrinsically involves complex signaling pathways [1]. The identification of dysregulated genes involved in the carcinogenesis and tumor progression as well as their control poses challenges that mobilize the cancer research community worldwide. High-throughput technologies now allow genome-wide expression profiling, which is already providing important insights into complex regulatory networks, enabling the identification of new or under-explored biological processes, and helping to uncover the genes that are involved in various pathological processes as is the case with cancer [2, 3]. Highly sensitive investigative transcriptome profiling is now carried out by *high throughput sequencing* (HTS). However, because of reduced cost, clinical diagnoses rely on a set of target genes (demonstrated to be relevant in the case analyzed in a previous investigative step) and, thus, involve *quantitative Real*-*Time RT*-*PCR* (qRT-PCR) or AmpliSeq [4]. In this context, qRT-PCR has already been incorporated into clinical and translational science practice as a result of redefining the classification criteria of breast tumor diagnosis and prognosis by the incorporation of molecular factors in state-of-the-art protocols [5–8]. The successful transfer of knowledge from basic research to clinical diagnosis necessarily involves the demonstration that the results obtained with the latter are statistically consistent with those obtained with the former.

Statistical consistency involves experimental reproducibility and, from a general viewpoint, reproducibility is an absolute prerequisite for reliable inference, especially when investigating the biological significance of subtle differences in gene expression [9]. Experimental reproducibility is generally linked to the concept of *robustness* that is understood as the stability of a system output (here, the gene expression) with respect to stochastic perturbations. When comparing data from one transcriptome profile to another, one performs normalization of gene expression at the level of sequence and sample sizes. The process of normalization itself increases the robustness of an inference drawn from an experiment because it decreases intra- and inter-sample variances. Cancer is a multifactorial disease whose dimensionality (understood in terms of the relevant parameter space) may vary in time and space. Thus, internal controls with the highest possible robustness of gene expression are necessary to compare independent experiments and to maximize the confidence of inferences drawn from independent assays. In terms of gene expression, the genes with the highest level of expression stability (or expression robustness) over time and space are called *housekeeping genes* (HKG), simply because these genes perform functions that are essential to any cells in any states. The main concept associated with HKGs when dealing with transcriptome profiling is the notion that their expression level should not: (i) be affected under pathological conditions, (ii) differ between tissues and cell types, and (iii) be altered in response to experimental treatments. As a consequence, HKGs are generally regarded as the best gene candidates for internal controls when comparing transcriptome profiles obtained independently. Thus, the choice of HKGs is essential to the success of the experiment performed, especially when transcriptome profiling is carried out on the basis of high throughput sequencing, where any differences of gene expression may have significant meaning according to the expression robustness of reference genes (the HKGs) [10–13].

In a previous study, we described a strategy for the selection of protein targets suitable for drug development against neoplastic diseases taking the case of breast cancer (BC) as a particularly pertinent example [14]. We extracted the sub-networks of down- and up-regulated human genes by comparing malignant and control cell lines and identified proteins that act as connectivity hubs representing suitable targets for disease control in terms of pharmacological agents. Surprisingly, this analysis revealed that the most frequently used *traditional* HKGs (tHKGs) such as GAPDH, ACTB and TUBA1A appeared significantly altered in their expression level from one sample to the other, which raises significant concerns regarding their uses as internal controls. To address this issue, we propose a strategy to identify potential *novel* HKGs (nHKGs) and also to validate tHKGs that may serve as internal controls in BC investigations based on HTS and qRT-PCR. First, we identified the genes with the highest level of expression stability in transcriptome data, and second, we confirmed that these genes were effectively the most stably expressed in qRT-PCR experiments of mRNA extracted from axenic cultures of the same cell lines. In cancer research, only a few studies attempted to investigate the variation of HKGs’ expression rates over different tissues and samples. Here, we used transcriptome and microarray data available from the ICGC consortium, TCGA and GEO to assess nHKG and tHKG candidates over different breast cancer tissue samples. We identified CCSER2, SYMPK, ANKRD17 and PUM1 as the top-four best candidates of HKGs for BC.

## Methods

### Interactome data

The protein connectivity inferences described below are based on the protein interactions given in the file intact-micluster.zip available from ftp://ftp.ebi.ac.uk/pub/databases/intact/current/psimitab/ (accessed on 04.04.2014) as described by Carels et al. [14]. Briefly, our resulting file contained 308,314 protein pairs. This interaction file was then processed to form a non-redundant list of Uniprot identifiers (UID) used to retrieve the corresponding protein sequences (68,504) by querying UniprotKB at http://www.uniprot.org/help/uniprotkb. The equivalence between UID and human genes was obtained by homology search (tBLASTn) of protein sequences (68,504) found as queries and human coding sequences (CDS) used as subjects from the dataset (hs37p1.EID.tar.gz) of Fedorov’s laboratory (available at http://bpg.utoledo.edu/~afedorov/lab/eid.html) [15]. Homologies were considered significant when their score was ≥120, E-value ≤10^−4^ and identity rate ≥80 % over ≥50 % of query size.

### Transcriptome data

We recovered transcriptome datasets of breast cell lines (MCF10A, BT-20, BT-474, MDA-MB-231, MDA-MB-468, MCF-7, T-47D, ZR-75-1, see information at http://www.atcc.org/) from http://www.illumina.com/science/data_library.ilmn. We retrieved 433 transcriptome datasets relative to breast cancers from the ICGC portal. All raw data analyzed can be accessed and downloaded via the ICGC data portal (http://dcc.icgc.org/). The data samples were generated from patients that presented distinct histological subtypes, ages, tumor stages and sizes, grades and menoposal status, in order to perform a blind validation experiment. Additionally, we retrieved 95 paired transcriptome datasets relative to BC and their non-tumoral samples from TCGA (http://cancergenome.nih.gov/), considering Luminal A, Luminal B, Triple Negative and HER2+. The gene expression profiles for cell lines and tumors were assessed through a homology search with the human CDS sample of the Fedorov laboratory. The sequences from transcriptome tags were used as queries in searches for the best homologies (BLASTn) with human CDSs. The homology redundancy in the BLASTn output file gave us the tag count per gene i.e., a profile of human gene expression for each sample considered. Homologous hits were considered significant when covering at least 50 % of their size.

Each gene expression profile (tag count per gene) was normalized according to the CDS size and whole tag count using the formula (10^9^*C)/(N*L), where 10^9^ is a correction factor, *C* is the number of reads that match a gene, *N* is the total number of mappable tags in the experiment, and *L* is the CDS size [16]. When tags were counted for more than one gene isoform (alternative splicing forms), we cumulated counts and allocated them to just one form (the largest one); this strategy means that we looked for gene expression and not isoform expression. To allow the comparison between independent gene expression profiles, we further applied Quantil-normalization (*Q*-norm) [17]. The normalization of tag samples according to the CDS size and tag number is necessary to avoid values of gene expression that may differ from one sample to the other. The distribution of tag counts from transcriptome data is typically a decreasing curve where the lowest expressed genes are the most frequent ones. The size of the human transcriptome used was 4379 genes common to the eight cell lines investigated in our experiment.

### Microarray analysis

We retrieved three microarray datasets of breast cancer (GSE9574, GSE20437 and GSE6434) from the Gene Expression Omnibus (GEO) repository (http://www.ncbi.nlm.nih.gov/geo/). GSE9574 includes 29 samples from histologically normal micro-dissected breast epithelium with 14 samples from epithelium adjacent to a breast tumor and 15 samples obtained from patients undergoing reduction mammoplasty without apparent breast cancer. GSE20437 includes 42 samples from laser capture micro-dissection (LCM) of normal breast tissue samples analyzed with the Affymetrix HU133A microarrays to show that histologically normal epithelium from breast cancer patients and cancer-free prophylactic mastectomy patients share a similar expression profile. Among these 42 samples (i) 36 were from the same age group with 18 from reduction mammoplasty and 18 from histologically normal epithelial samples of breast cancer patients from which 9 were ER+ and 9 ER- and (ii) 6 were histologically normal epithelial samples from prophylactic mastectomy patients. GSE6434 includes 24 BC patients sensitive or resistant to docetaxel that were analyzed with the Affymetrix Human Genome U95 Version 2 Array.

### HKGs

**Fig. 1:**
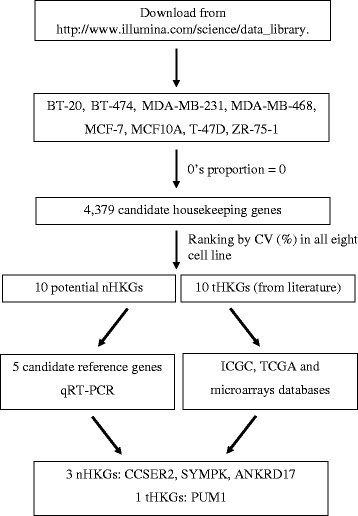
Flowchart of a novel identification strategy for housekeeping genes (nHKGs) in breast cancer. A list of 7780 genes was first obtained by selecting genes with low *0’s proportion* and coefficient of variation (CV). 0’s proportion is defined as the proportion of different cell lines in which a given gene is not expressed; and CV is the ratio of the standard variation to the mean. Among the nHKG candidates, 10 with the lowest CVs were further validated by qRT-PCR and for their 0’s proportion in a large human tissue dataset from ICGC. For the sake of comparison, we included traditional housekeeping genes (tHKGs) selected from the literature, i.e., PUM1, RPL13A, PGK1, GUSB, ACTB, DIMT1, TUBA1A, GAPDH, B2M, 18S

We selected 10 tHKGs among the genes most commonly used as internal control in expression experiments to evaluate their expression variance by HTS and qRT-PCR (see Table 1 for gene name, uniprotkb, function; and see Additional file 1: Table S1 for primer sequence). The strategy used to identify nHKGs is outlined in Fig. 1. We searched for candidate HKGs whose expression was detected in the transcriptomes of eight cell lines (MCF10A, BT-20, BT-474, MDA-MB-231, MDA-MB-468, MCF-7, T-47D, ZR-75-1) using *0’s proportion* in transcriptome datasets. 0’s proportion is defined as the proportion of different cell lines in which a given gene is not expressed and was calculated as follows:

**Table 1:** Features of nHKGs and tHKGs

Uniprotkb	Protein name	Gene	Mean	CV (%)	Biological process
nHKGs					
Q92575	UBX domain-containing protein 4	UBXN4	81.75	11.45	Response to unfolded protein
Q08211	ATP-dependent RNA helicase A	DHX9	158.25	12.42	ATP catabolic process, DNA duplex unwinding
P17152	Transmembrane protein 11, mitochondrial	TMEM11	68.12	12.83	Mitochondrion organization
Q6PKG0	La-related protein 1	LARP1	137.87	13.10	Cell proliferation
Q13190	Syntaxin-5	STX5	65.37	13.33	Vesicle transport
O75179	Ankyrin repeat domain-containing protein 17	ANKRD17	33.62	14.03	Blood vessel maturation
Q92797	Symplekin	SYMPK	60.75	14.29	Cell adhesion
Q6P1X5	Transcription initiation factor TFIID subunit 2	TAF2	23.87	14.41	G2/M transition of mitotic cell cycle
Q9H7U1	Serine-rich coiled-coil domain-containing protein 2	CCSER2	20.00	15.12	Microtubule bundle formation
Q6NZ67	Mitotic-spindle organizing protein 2B	MZT2B	179.62	15.69	–
tHKGs					
Q14671	Pumilio homolog 1	PUM1	45.50	24.56	Vesicle-transport, translation
P40429	60S ribosomal protein L13a	RPL13A	3028.62	25.51	Translation
P00558	Phosphoglycerate kinase 1	PGK1	488.37	30.91	Glycolysis
P08236	Beta-glucuronidase	GUSB	70.00	36.56	Metabolic processes
P60709	Actin, cytoplasmic 1	ACTB	9500.25	37.06	Protein folding, chromatin remodeling
Q9UNQ2	Probable dimethyladenosine transferase	DIMT1	26.62	41.82	rRNA processing
Q71U36	Tubulin alpha-1A chain	TUBA1A	658.00	47.50	Protein folding, G2/M transition of cell cycle
P04406	Glyceraldehyde-3-phosphate dehydrogenase	GAPDH	3580.12	66.79	Metabolic process, protein folding
P61769	Beta-2-microglobulin	B2M	1530.12	93.47	Immunity

1 $$ 0'\mathrm{s}\ \mathrm{proportion}=\frac{\mathrm{Number}\ \mathrm{of}\ \mathrm{cell}\ \mathrm{lines}\ \mathrm{not}\ \mathrm{expressing}\ \mathrm{the}\ \mathrm{gene}}{\mathrm{Total}\ \mathrm{number}\ \mathrm{of}\ \mathrm{cell}\ \mathrm{lines}} $$

A 0 value for the 0’s proportion indicates that the gene is expressed in the eight cell lines, and a value between 0 and 1 indicates that the gene is not expressed in at least one cell line.

To select nHKGs, we (i) eliminated the genes that were not-expressed in all cell lines (0’s proportion =1) from the list; (ii) evaluated the coefficient of variation (CV) for each gene, which is the ratio of the standard variation and the mean; (iii) further filtered out potential nHKGs by keeping the 10 genes with the lowest CV among the 4379 genes common to the eight cell line transcriptomes (tumoral and non-tumoral cell lines).

In order to annotate HKGs, we searched for their homologies with *nr* (GenBank, rel 181) using the BLAST to gene ontology - Blast2GO [18]. We also looked for the most common transcription factors (TFs) involved in BC signaling pathways that could regulate HKG expression by searching the literature, and selected the following ones: AP1, NFKB, GATA3, FOXA1, ER, Elk1, STAT3, STAT5, HIF, NOTCH, SP1, TP53, MYC [19]. In order to crosscheck the information available as far as possible, we also compared our data with three reference databases: (i) STRING (http://string-db.org/), which includes direct and indirect associations derived from four sources: genomic context, high-throughput experiments, (conserved) co-expression and previous knowledge, (ii) CCSB interactome (http://interactome.dfci.harvard.edu/) and (iii) cancer-systemsbiology (http://www.cancer-systemsbiology.org/). In order to determine the degree of interdependence associated to HKGs, we graphically analyzed their sub-networks formed with TFs in the GEPHI (http://gephi.github.io/) environment by pasting data in the input node file and using the toolbox of this program to automatically calculate and represent protein connectivity (i.e., the relative number of edges per node).

### Cell culture, cDNA preparation and qRT-PCR

To validate our *in silico* inferences, we used four breast tumoral cell lines: MCF-7 (Luminal A), T47D (Luminal A), MDA-MB-231 (Triple Negative), MDA-MB-468 (Triple Negative), and a non-tumoral breast cell line, MCF-10A. All cell lines were cultured in standard conditions as recommended by ATCC, supplemented with 10 % fetal bovine serum (FBS), 100 IU/ml penicillin and 100 mg/ml streptomycin in a humidified environment containing 5 % CO_2_ at 37 °C.

We isolated total RNA from breast cell lines using a PureLink RNA Mini Kit (Ambion) according to the manufacturer’s instructions. Total RNA was eluted in 40 μl of RNase-free H_2_O and stored at −80 °C. Extracted RNAs were quantified using NanoDrop ND-1000 (NanoDrop Technologies) and the absorbance ratios at 260/280 and 260/230 were measured to assess RNA purity. The ratios of optical densities (OD) at 260 vs. 280 nm (260/280) were between 1.8 and 2.0 for all samples. First-strand cDNA synthesis was carried out with 1 μg total RNA using oligo(dT) primers and Superscript II reverse transcriptase (Invitrogen Life Technologies) following manufacturer’s instructions. PCR assays were performed using the primers listed in Additional file 1: Table S1. All oligonucleotides were analyzed for potential secondary structure and dimerization using OligoAnalyzer 3.1. qRT-PCR was performed on a StepOne Plus System (Applied Biosystems) using Power SYBR Green PCR Master Mix (Applied Biosystems). PCR was done using the following protocol: 50 °C for 2 min, initial denaturation 94 °C for 5 min, then 40 cycles at 94 °C for 30 s, 60 °C for 30 s, 72 °C for 45 s; and 72 °C for 15 min. To verify that the used primer pair produced only a single product, a DNA melting curve analysis was added after thermocycling, determining dissociation of the PCR products from 60 to 90 °C (with a heating rate of 0.2 °C and continuous fluorescence measurement). The amplification efficiency of each set of oligonucleotides was determined by plotting the cycle threshold (Ct) values obtained for four cDNA dilutions (1:100, 1:200, 1:400, 1:800) (Additional file 2: Figure S1).

## Results

### Identification of HKGs from transcriptome data

Table 1 shows the list of top-10 candidates of nHKGs obtained from the analysis of the eight breast cell lines selected. Among genes with low CV (%) values across breast cell lines, some may have either a low or a large average expression level. Because of their ease of detection, the HKGs with large average expression levels are suitable for gene expression characterization by RT-PCR, microarrays and/or HTS. The top-10 nHKGs (DHX9, MZT2B, UBXN4, LARP1, TAF2, CCSER2, STX5, SYMPK, TMEM11 and ANKDR17) with the smallest expression variability identified here have not been used yet as internal control in expression experiments and have independent functions in cellular maintenance (Table 1). Interestingly, GAPDH, ACTB and TUBA1A, the most commonly reported reference genes for comparative expression experiments, did not meet the parameters applied by us for the selection of nHKGs. However, for the sake of comparison, we included the nine tHKGs most commonly found in the literature (PUM1, RPL13A, PGK1, GUSB, ACTB, DIMT1, TUBA1A, GAPDH and B2M). The tHKGs did not belong to the list of top-100 genes with the lowest coefficient of variation (the standard deviation over the average of a random variable) of gene expression.

**Fig. 2:**
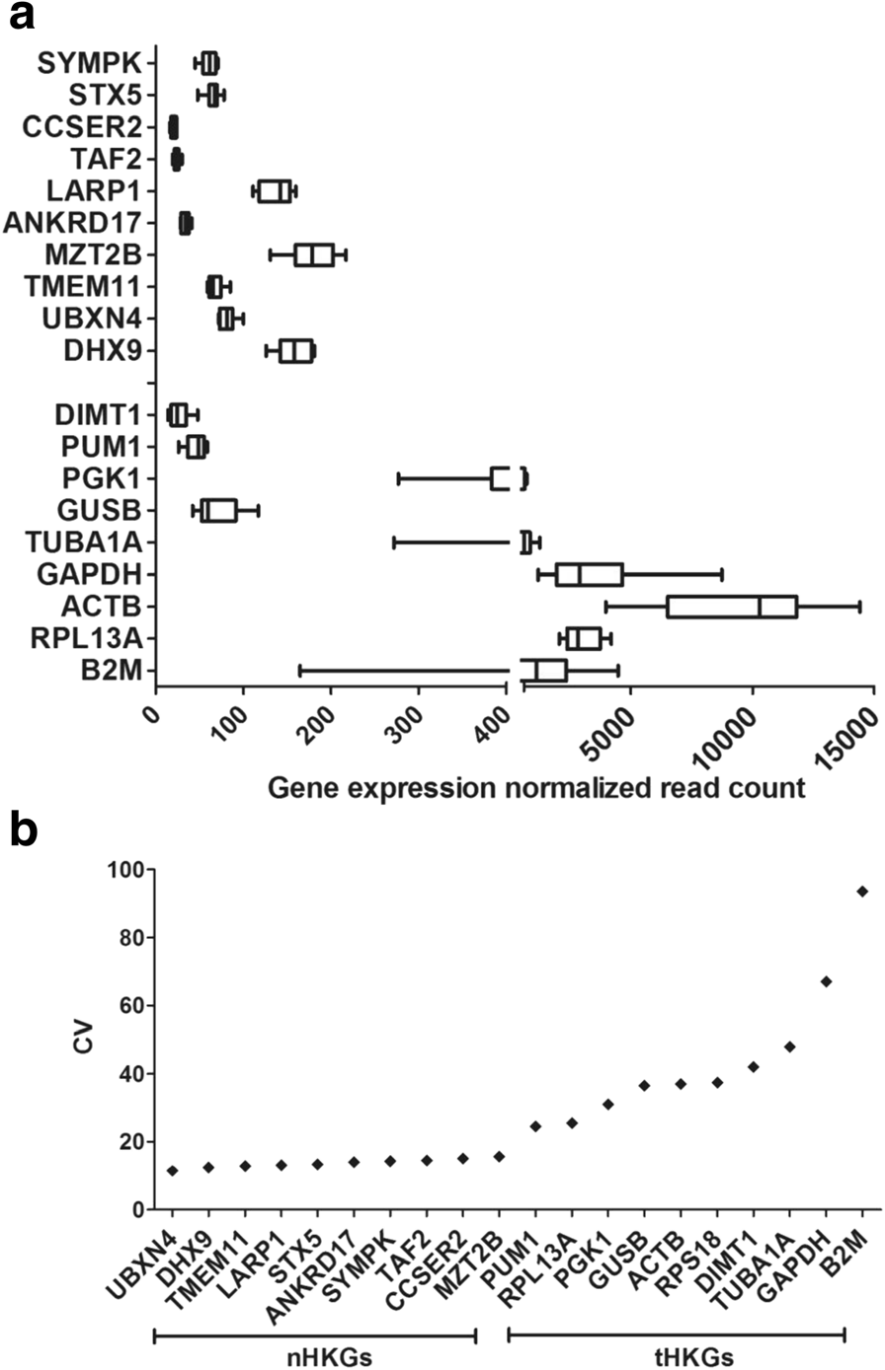
Pattern and variation of candidate nHKG and tHKG expression in breast cell lines. **a** Comparison of gene expression between nHKG candidates and tHKGs across all cell lines. The *x*-axis represents the mean gene expression levels. Boxes delimit lower and upper quartiles while the vertical lines within the boxes indicate median expression values. Lateral whiskers provide lower and higher values as left and right ticks, respectively. **b** Housekeeping genes (nHKGs and tHKGs, *x*-axis) ordered by an increasing average of CVs for each gene over eight cell lines (*y*-axis) across breast cell lines

The average expression level of nHKGs is 82.92 (for a range of 20.00 to 179.62), and that of tHKGs is 2323.87 (range of 26.62 to 9500.25) (Fig. 2a and Table 1). The average expression of nHKGs was an order of magnitude lower than that of tHKGs. Figure 2b shows the CV for each gene over the eight breast cell lines. tHKGs shows a ~10 time larger CV (44.91, on average) than nHKGs (3.67, on average), supporting the notion that nHKGs are generally expressed more stably and at lower levels than tHKGs.

**Fig. 3:**
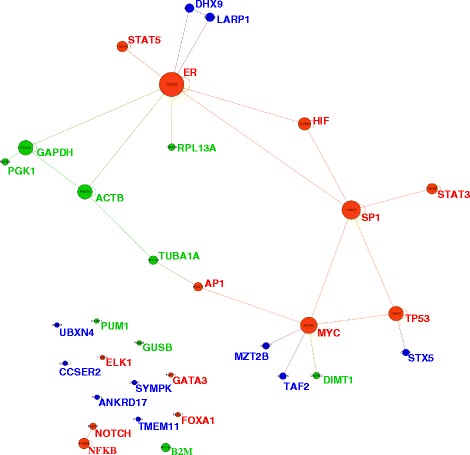
Subnetworks of nHKGs, tHKGs and transcription factors in Gephi. Nodes are for genes and links for interactions among them. Node size indicates connectivity grade. Red is for transcription factors, green for tHKGs, and blue for nHKGs

For the purpose of challenging the robustness of nHKG to expression variation, we tested the co-regulation between HKGs by examining the network of transcription factors involved in BC signaling pathways in nHKGs as well as in tHKGs (Fig. 3 and Additional file 3: Figure S2). The interpretation of resulting networks in the light of the version of human interactome that we used provides evidence that estrogen receptor (ER) directly regulates DHX9, LARP1, ACTB, GAPDH and RPL13A expression; whereas MYC regulates DIMT1, MZT2B and TAF2; TP53 regulates STX5; and AP1 regulates TUBA1A. We also found interactions between GAPDH, PGK1, ACTB and TUBA1A; and also between DHX9 and LARP1, indicating that these genes present regulation processes that share common routes. These interactions are also reported in STRING (Additional file 3: Figure S2), CCSB (Additional file 4: Table S2) and cancer-systemsbiology databases (Additional file 5: Table S3). By contrast, according to the common knowledge available at present, CCSER2, TMEM11, SYMPK, UBXN4 and ANKRD17 among nHKGs do not show cross interactions. Thus, CCSER2, TMEM11, SYMPK, UBXN4 and ANKRD17 represented the best candidates for potential nHKGs offering internal control in comparative expression assays. By assessing the relationship between the expression patterns of nHKGs and tHKGs using the STRING database, we found that, with the exception of DHX9 that exhibits positive correlation with UBXN4, all remaining nHKGs are independently expressed (Additional file 6: Figure S3A). Among tHKGs, GAPDH shares expression with PGK1, ACTB and TUBA1A and the expression of TUBA1A is directly associated with ACTB (Additional file 6: Figure S3B). Based on all these results taken together, we selected CCSER2, TMEM11, SYMPK, UBXN4 and ANKRD17 as a set of nHKGs for validation. These nHKGs include genes with the largest expression stability among the transcriptome data of our cell line sample as well as the absence of obvious co-regulation and co-expression with other genes. Additionally, we selected TUBA1A, GAPDH, ACTB, B2M and 18S as a set of tHKGs for comparison.

### Evaluation of selected nHKGs and tHKGs by qRT-PCR

**Fig. 4:**
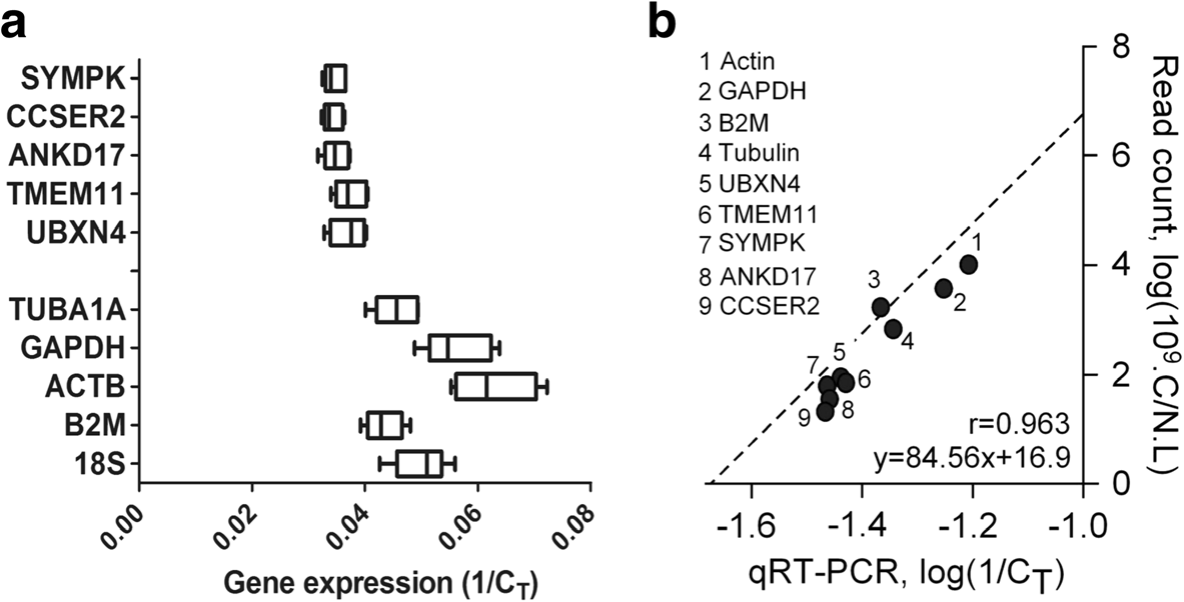
Scatter plot of gene expression measured by real-time PCR and read counting in RNA-seq data. **a** Expression pattern of nHKG candidates and tHKGs in breast cell lines obtained by qRT-PCR across eight cell lines. Boxes delimit lower and upper quartiles while the vertical lines within the boxes indicate median expression values. Lateral whiskers provide lower and higher values as left and right ticks, respectively. The *x*-axis gives the mean of 1/C_T_ for each gene. Results are representative of three independent experiments. **b** Correlation between qRT-PCR and read count from RNA-seq data for each gene across eight breast cell lines (*r* = 0963). The *y*-axis represents the average of CVs for each gene over eight cell lines

To validate inferences about nHKGs and tHKGs from HTS data, we next performed qRT-PCR analyses with the five nHKG candidates (CCSER2, SYMPK, TMEM11, UBXN4 and ANKDR17) for comparison with tHKG expression (ACTB, GAPDH, TUBA1A, B2M and 18S). In order to compare our results across cell lines and genes, we used the simplest and most direct method, the comparative C_T_ method, which uses only raw values of threshold cycles C_T_. Since C_T_ is inversely proportional to the gene expression, we compared gene expression according to 1/C_T_ as shown in Fig. 4a. The amplification of cDNA with gene-specific primers from an independent set of breast cell lines (MCF-10A, MCF-7, MDA-MB-231, MDA-MB-468 and T47D) demonstrated better performance in terms of stability for nHKGs than tHKGs (Fig. 4a and Table 2). nHKGs showed nearly the same gene expression levels for each sample of the whole set (Fig. 4a). Based on qRT-PCR data, the average expression of nHKGs was 28.18 when calculating C_T_ (σ_CT_ = 0.94) and 35.66 10^−3^ (σ_1/CT_ = 1.2 10^−3^) when calculating 1/C_T_ (C_T_ ranging from 29.23 to 26.82 or 1/C_T_ ranging from 34 10^−3^ to 37 10^−3^) and that of tHKGs was 19.86 (σ_CT_ = 2.31) and 51.66 10^−3^ (σ_1/CT_ = 6.4 10^−3^) when calculating 1/C_T_ (C_T_ ranging from 23.15 to 16.07 or 1/C_T_ ranging from 43 10^−3^ to 63 10^−3^). Furthermore, for HTS data, the average expression of nHKGs was lower than that of tHKGs. Additionally, we confirmed that the CV of nHKGs (6.63 on average for C_T_ and 52.72 10^−3^ for 1/C_T_) was lower than that of tHKGs (9.74 on average for C_T_ and 73.62 10^−3^ for 1/C_T_) (Fig. 4a and Table 2). All these results support our previous hypothesis that nHKGs are generally expressed more stably and at lower levels than tHKGs. By comparing the logarithm of average normalized read counts of RNA-seq data to the logarithm of 1/C_T_ values of qRT-PCR for each gene, we observed a strong linear correlation (*r* = 0.963), which allows the transposition of results obtained with one technique to the other and *vice versa* (Fig. 4b).

**Table 2:** Threshold cycle (C_T_): Values of average, standard deviation and coefficient of variation for tHKGs and nHKGs

C_T_	GAPDH	B2M	ACTB	TUBA1A	18S	CCSER2	UBXN4	SYMPK	TMEM11	ANKRD17
MCF-7	18.51	23.97	16.24	21.92	20.49	29.72	26.61	29.46	27.04	28.82
MDA-MB-231	16.45	20.80	13.84	20.22	19.61	27.47	24.83	27.35	24.69	27.12
MDA-MB-468	20.48	25.49	18.09	24.94	23.47	30.96	30.49	30.84	29.44	31.57
T47D	15.66	22.14	14.62	20.32	19.56	28.01	25.33	27.34	24.94	26.80
MCF10A	18.29	23.32	17.56	22.69	17.86	29.96	28.61	29.74	27.98	29.25
Minimum	15.67	20.81	13.85	20.23	17.87	27.47	24.84	27.35	24.69	26.81
Maximum	20.49	25.50	18.10	24.94	23.47	30.96	30.50	30.84	29.45	31.57
Mean	17.88	23.15	16.07	22.02	20.20	29.23	27.18	28.95	26.82	28.72
Std. Deviation	1.892	1.782	1.831	1.942	2.062	1.444	2.359	1.550	2.021	1.912
Coefficient of variation (%)	10.58	7.70	11.39	8.82	10.21	4.94	8.68	5.35	7.53	6.66

Good laboratory practice would recommend the use of more than one internal control for comparative analyses of gene expression to minimize the risk associated with accidental errors and to increase statistical consistency. Thus, we assessed the potential combination of all five genes based on qRT-PCR data. Correlation coefficients (r) were calculated, representing the relationship between the expression of each individual housekeeping gene and the mean expression of the remaining genes (Table 3). Clearly, all nHKGs showed a very high correlation coefficient, which means that we could alternatively choose and combine each one of nHKGs. By extension, if three nHKGs are used as internal controls, at least two should give similar levels of gene expression in order to provide confidence in the experimental results obtained.

**Table 3:** Correlation coefficients for the expression of each individual gene and the mean expression of the remaining four genes

	CCSER2	UBXN4	SYMPK	TMEM11	ANKD17
MCF-7	29.72351	26.61776	29.46543	27.04637	28.82998
MDA-MB-231	27.4733	24.83932	27.3512	24.69488	27.12572
MDA-MB-468	30.9613	30.49971	30.84297	29.44699	31.57093
T47D	28.0175	25.33814	27.34727	24.94886	26.80775
MCF10A	29.96375	28.61279	29.74568	27.98631	29.25333
Correlation	0.976768	0.969011	0.983436	0.99814	0.977064

### Validation of nHKGs and tHKGs in large breast cancer tissue datasets from ICGC, TCGA and GEO

We obtained the transcriptome expression patterns of 433 tissue samples associated with breast cancer from the ICGC consortium, 95 paired tissue samples from TCGA, and three distinct microarray datasets from GEO and successively screened these data for nHKGs and tHKGs validation. This assay presented three main goals: (i) validation of nHKGs for use in clinical conditions, (ii) generalization of the nHKG and tHKG expression data obtained with malignant breast cell lines to human breast tumors, and (iii) assessment of tHKGs expression variability in malignant tissues of human breast.

**Fig. 5:**
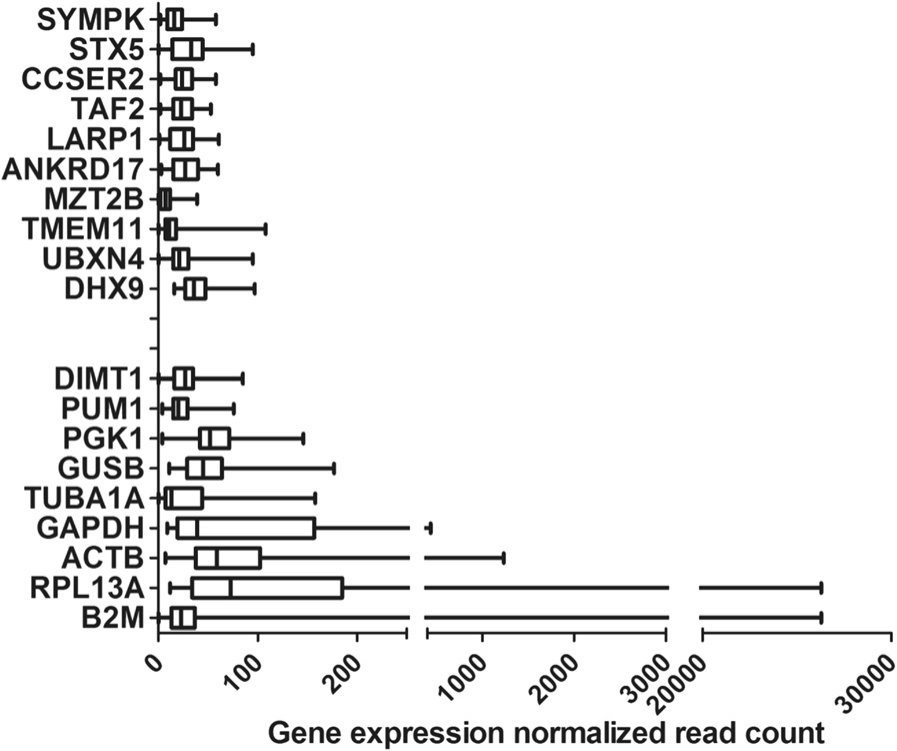
Distribution of expression levels by read counts of 10 nHKGs and 9 tHKGs in breast cancer RNA-seq (*n* = 433) from ICGC. Boxes delimit lower and upper quartiles while the vertical lines within the boxes indicate median expression values. Lateral whiskers provide lower and higher values as left and right ticks, respectively

The expression levels of DHX9, LARP1, TAF2, CCSER2, SYMPK, and ANKDR17 (nHKGs) were measurable in all 433 samples, while we did not observe expression of MZT2B, UBXN4, STX5 and TMEM11 in all samples. These results mean that among the 5 core nHKGs, we identified CCSER2, SYMPK and ANKDR17 as the best nHKG candidates. It is worth stressing that among tHKGs, DIMT1, TUBA1A and B2M were not expressed in all samples. However, PUM1 appeared to be the best tHKG candidate for use as internal controls in BC investigations. Comparison of the mean expression levels, standard deviations and CV of tissue sample sets revealed that nHKGs showed lower values than the commonly used housekeeping genes (Fig. 5). The average expression of nHKGs by RNA-seq read count was 24.97 and that of tHKGs was 247.66 (Table 4). With respect to expression variation, most of the tHKGs showed relatively higher variation levels than the majority of nHKGs and the mean CV values of nHKGs (55.94, on average) was ~3 times lower than those of tHKGs (161.39, on average) (Table 4). Similar results were also observed when we analyzed TCGA and microarray data (Additional file 7: Figure S4 and Additional file 8: Figure S5). All these data support the hypothesis that nHKGs are generally expressed more stably and at lower levels than tHKGs. The results of our data analysis identified CCSER2, SYMPK, ANKDR17 and PUM1 as suitable reference genes for bench experiments of gene expression since they showed low variation, but persistent expression across individual tissues and over large-scale sampling. Given that the expression stability of nHKGs was consistently better than that for the majority of tHKGs, one may conclude here that nHKGs are better internal controls than tHKGs to report on disease and/or tissue-specific effects on the basis of molecular investigations. Our results indicate CCSER2, SYMPK, ANKRD17 and PUM1 to be the best HKG candidates for clinical and *in vitro* investigations in BC.

**Table 4:** Expression level, average, standard deviation, median and coefficient of variation values of nHKGs and tHKGs in a large data set of breast cancer tumors (*n* = 433) from ICGC

	Minimum	25 % percentile	Median	75 % percentile	Maximum	Mean	Std. deviation	Coefficient of variation (%)
DHX9	16.00	27.00	36.00	47.50	97.00	39.22	14.56	37.14
MZT2B	0.0	2.000	7.000	12.00	39.00	7.896	6.834	86.55
UBXN4	0.0	15.00	21.00	30.50	95.00	23.94	12.93	54.04
ANKRD17	3.000	15.00	27.00	40.00	60.00	27.55	13.88	50.37
LARP1	1.000	12.00	26.00	35.00	61.00	25.53	13.92	54.54
TAF2	2.000	15.00	23.00	34.00	53.00	23.94	11.95	49.94
CCSER2	2.000	17.50	24.00	34.00	58.00	25.58	11.64	45.50
STX5	0.0	14.00	33.00	44.50	95.00	32.82	21.33	65.00
TMEM11	0.0	7.000	11.00	18.00	108.0	14.10	12.07	85.62
SYMPK	2.000	9.000	16.00	24.00	58.00	17.40	10.58	60.80
PUM1	4.000	15.00	20.00	29.50	76.00	22.63	11.32	50.03
DIMT1	0.0	16.00	27.00	35.00	85.00	27.93	15.46	55.38
ACTB	7.000	37.50	59.00	102.5	1232	87.07	108.7	124.8
GUSB	11.00	29.00	45.00	64.00	177.0	53.06	32.19	60.67
GAPDH	9.000	19.00	39.00	157.0	437.0	97.21	98.85	101.6
TUBA1A	0.0	7.000	13.00	44.00	158.0	26.07	24.98	95.80
B2M	0.0	13.00	23.00	37.00	26248	608.1	3264	536.73
RPL13A	12.00	34.00	73.00	185.0	26248	1094	4564	417.25
PGK1	4.000	42.00	52.00	71.50	146.0	59.78	29.30	49.02

## Discussion

Despite the considerable progress in high-throughput technologies, a rational method design to identify HKGs has not been achieved yet. Until now, no fully effective reference HKGs have been proposed for comparative analyses of gene expression in the context of complex diseases, such as cancer, neurological, autoimmune, cardiovascular and metabolic diseases. Such lack of critical assessment can promote biases in the conclusions drawn from these investigations. Thus, we believe that the strategy that we outlined here is relevant for the identification of suitable HKGs as internal control for bench experiments on gene expression in BC, and should be explored for other neoplasias and diseases.

Our findings illustrate the importance of minimizing any sources of bias and suggest the importance of critically assessing the performance of the HKGs used as internal controls in each case studied. We used transcriptome data to select genes with low variability in expression levels across breast cell lines. Our large-scale dataset samples were filtered out to identify genes with the largest expression stability across breast cell lines. Further screening including the elimination of candidate genes with obvious co-regulation, co-expression and/or similar biological function was successfully added to the protocol. HKGs distributed within different functional classes significantly reduce the chance of genes co-regulation. All these criteria taken together increase the likelihood of independent expression of candidate HKGs and decrease the likelihood of expression alterations in the context of complex networks such as those found in cancer diseases.

Clearly, the use of nHKGs is expected to improve the robustness likelihood of bench experiments aimed to validate bioinformatic inferences in the context of BC for *in vitro* models. We demonstrated a very high correlation level (*r* = 0.963) between expression levels obtained from RNA-seq data (Illumina sequencing) and qRT-PCR using the same cell lines despite being cultured in a different place, at a different time, on different media and from independent sources; a set of modifications that represents a huge source of potential variability. The high correlation level and the almost perfect match with the linear regression of RNA-seq and qRT-PCR data gives a simple mean for direct result extrapolation from one result to another. As a consequence, a real possibility exists to translate the expression data of investigative RNA-seq into diagnosis at a clinical level by using qRT-PCR or AmpliSeq. Such a high level of robustness of gene expression on a multidimensional scale suggests that CCSER2, ANKRD17 and SYMPK are suitable nHKGs as well as the tHKG PUM1 for fine comparative analyses of gene expression by HTS and qRT-PCR.

Most of the tHKGs selected here have been indiscriminately used by a number of scientists worldwide and are available commercially as standard kits. Typically, these kits focus on a specific pathway and include a panel of genes relevant to that specific pathway or disease state. For example, the cancer-pathway kit from Qiagen array includes: B2M, HPRT1, RPL13A, GAPDH, ACTB while that of Life technologies array includes: CDKN1B, G6PD, POLR2A, IPO8, CASC3, YWHAZ, CDKN1A, UBE2D2, HMBS, UBC,TP5B, HPRT1, CUL1, 18S, RPLP0, ACTB, PPIA, GAPDH, PGK1, B2M, GUSB, HPRT1, TBP, TFRC. On the other hand, ACTB, GAPDH, RPLP0, GUSB and TFRC form a set of reference genes included in a commercial Oncotype DX test. This test was supported by the National Comprehensive Cancer Center Network (NCCN) and the American Society of Clinical Oncology (ASCO) in their treatment guidelines [20] in order to calculate a recurrence risk score for each patient. Here, we have shown that most of these genes are not stably express across breast cell lines. As a result, in a large subset of human tissues, the introduction of these genes as reference HKGs is expected to promote noise in the assessment of expression levels from other genes. As a matter of fact, this situation can be expected since tHKGs have a higher level of connection with other genes, such as TFs for example, than nHKGs.

Astounding discrepancies can be found in the data from the literature when considering the most frequently used tHKGs in qRT-PCR as internal controls. Révillion et al. [21] showed an association of GAPDH expression with BC cell proliferation and with the aggressiveness of tumors. Ahmad et al. [22] demonstrated phosphoglycerate kinase 1 (PGK1) as a promoter of metastasis in colon cancer. Hence, PGK1 is a promoting enzyme for peritoneal dissemination in gastric cancer [23]. McNeill et al. [24] showed alterations in GUSB expression in breast cancer. Stromal myofibroblasts in invasive breast cancer expression of alpha-smooth muscle actin (α-SMA) correlate with worse clinical outcomes [25] and the metastasis group showed significantly higher α-SMA expression compared with the non-metastasis group. Loss of α-tubulin was significantly correlated with distant metastases [26]. B2M expression demonstrated a significant difference in the breast cancer molecular subtypes, and may be related to apoptosis regulation in breast cancer [27].

The expression pattern of each nHKG selected here accurately reflected the mean expression pattern of the others. This demonstrates that the expression of each single nHKG is expected to be similar to the other four nHKGs, which is an important point in relation to the use of more than one HKG to normalize each assay and increase the assessment consistency. A universal internal control based on only one ideal HKG may not exist, thus we recommend to normalize bench experiments with a panel of HKGs whose expression has been proven to be as minimally variable as possible and the most robust as possible regarding variation under experimental conditions. In order to warrant robustness, the average of nHKG expression in one experiment should serve as internal control for comparison among experiments.

## Conclusions

In summary, we have modeled the performance of candidate HKGs to test their goodness-of-fit in serving as internal controls for comparative analysis of gene expression through HTS and qRT-PCR. A major advantage of a model approach is that the genes are placed within a robust bioinformatics and bench framework, which allows the strategy to be generalized to a variety of different diseases and cancer types.

## Additional files

Additional file 1: Table S1. Primer sequences for gene expression measure by qRT-PCR. (DOC 44 kb) Additional file 2: Figure S1. Standard curve and serial dilutions for nHKGs and tHKGs. The *x* axis represents the dilution series (1:800, 1:400, 1:200 and 1:100) and the *y* axis represents the mean of C_T_ for each gene. The correlation coefficient *r* is given for each gene inside parentheses. (PDF 53 kb) Additional file 3: Figure S2. Subnetworks of nHKGs, tHKGs and transcription factors from STRING in graph layout. Nodes are for genes and links for interaction among them. The color code for edge notation is given on the bottom left. (PDF 301 kb) Additional file 4: Table S2. Protein-protein interaction for tHKGs and nHKGs. Data from the CCSB interactome database. (DOC 128 kb) Additional file 5: Table S3. Protein-protein interaction for tHKGs and nHKGs. Data from the cancer-systemsbiology interactome database. (DOC 232 kb) Additional file 6: Figure S3. Co-expression of genes in the nHKG and tHKG groups as obtained from STRING. Color intensity represents the association score between each pair of gene nHKGs (A) and tHKGs (B). (PDF 193 kb) Additional file 7: Figure S4. Distribution of expression levels by normalized read counts of 10 nHKGs and 9 tHKGs in breast cancer RNA-seq (*n* = 95 paired samples) from TCGA. Boxes delimit lower and upper quartiles while vertical lines within boxes indicate median expression values. Lateral whiskers provide lower and higher values as left and right ticks, respectively. (PDF 371 kb) Additional file 8: Figure S5. Distribution of expression levels of 10 nHKGs and 9 tHKGs in microarrays of breast cancer samples from the GEO repository. Boxes delimit lower and upper quartiles while the vertical lines within the boxes indicate median expression values. Lateral whiskers provide lower and higher values as left and right ticks, respectively. (PDF 319 kb)

## References

[1] Hanahan D, Weinberg RA (2011). Hallmarks of cancer: the next generation. Cell.

[2] Kristensen VN, Lingjærde OC, Russnes HG, Vollan HK, Frigessi A, Børresen-Dale (2014). Principles and methods of integrative genomic analyses in cancer. Nat Rev Cancer.

[3] Do R, Stitziel NO, Won HH, Jørgensen AB, Duga S, Angelica MP (2015). Exome sequencing identifies rare LDLR and APOA5 alleles conferring risk for myocardial infarction. Nature.

[4] Yousem SA, Dacic S, Nikiforov YE, Nikiforova M (2013). Pulmonary Langerhans cell histiocytosis: profiling of multifocal tumors using next-generation sequencing identifies concordant occurrence of BRAF V600E mutations. CHEST.

[5] Wilson TR, Xiao Y, Spoerke JM, Fridlyand J, Koeppen H, Fuentes E (2014). Development of a robust RNA-based classifier to accurately determine ER, PR, and HER2 status in breast cancer clinical samples. Breast Cancer Res Treat.

[6] Tzovaras A, Kladi-Skandali A, Michaelidou K, Zografos GC, Missitzis I, Ardavanis A, Scorilas A (2014). BCL2L12: a promising molecular prognostic biomarker in breast cancer. Clin Biochem.

[7] Andres SA, Brock GN, Wittliff JL (2013). Interrogating differences in expression of targeted gene sets to predict breast cancer outcome. BMC Cancer.

[8] D’Cunha J, Maddaus MA (2006). The use of real-time polymerase chain reaction in thoracic malignancies. Thorac Surg Clin.

[9] Janssens N, Janicot M, Perera T, Bakker A (2004). Housekeeping genes as internal standards in cancer research. Mol Diagn.

[10] Kılıç Y, Çelebiler AÇ, Sakızl M (2014). Selecting housekeeping genes as references for the normalization of quantitative PCR data in breast cancer. Clin Transl Oncol.

[11] Kwon MJ, Oh E, Lee S, Roh MR, Kim SE, Lee Y (2009). Identification of novel reference genes using multiplatform expression data and their validation for quantitative gene expression analysis. PLoS One.

[12] Lee S, Jo M, Lee J, Koh SS, Kim S (2007). Identification of novel universal housekeeping genes by statistical analysis of microarray data. J Biochem Mol Biol.

[13] de Kok JB, Roelofs RW, Giesendorf BA, Pennings JL, Waas ET, Feuth T, Swinkels DW, Span PN (2005). Normalization of gene expression measurements in tumor tissues: comparison of 13 endogenous control genes. Lab Invest.

[14] Carels N, Tilli T, Tuszynski JA (2015). A computational strategy to select optimized protein targets for drug development toward the control of cancer diseases. PLoS One.

[15] Shepelev V, Fedorov A (2006). Advances in the Exon-Intron Database. Brief Bioinform.

[16] Mortazavi A, Williams BA, McCue K, Schaeffer L, Wold B (2008). Mapping and quantifying mammalian transcriptomes by RNA-Seq. Nat Methods.

[17] Bolstad BM, Irizarry RA, Astrand M, Speed TP (2003). A comparison of normalization methods for high density oligonucleotide array data based on variance and bias. Bioinformatics.

[18] Wang J, Yin Y, Hua H, Li M, Luo T, Xu L (2009). Blockade of GRP78 sensitizes breast cancer cells to microtubules-interfering agents that induce the unfolded protein response. J Cell Mol Med.

[19] Karamouzis MV, Papavassiliou AG (2011). Transcription factor networks as targets for therapeutic intervention of cancer: the breast cancer paradigm. Mol Med.

[20] Carlson JJ, Roth JA (2013). The impact of the Oncotype Dx breast cancer assay in clinical practice: a systematic review and meta-analysis. Breast Cancer Res Treat.

[21] Révillion F, Pawlowski V, Hornez L, Peyrat JP (2000). Glyceraldehyde-3-phosphate dehydrogenase gene expression in human breast cancer. Eur J Cancer.

[22] Ahmad SS, Glatzle J, Bajaeifer K, Bühler S, Lehmann T, Königsrainer I (2013). Phosphoglycerate kinase 1 as a promoter of metastasis in colon cancer. Int J Oncol.

[23] Zieker D, Königsrainer I, Tritschler I, Löffler M, Beckert S, Traub F (2010). Phosphoglycerate kinase 1 a promoting enzyme for peritoneal dissemination in gastric cancer. Int J Cancer.

[24] McNeill RE, Miller N, Kerin MJ (2007). Evaluation and validation of candidate endogenous control genes for real-time quantitative PCR studies of breast cancer. BMC Mol Biol.

[25] Yamashita M, Ogawa T, Zhang X, Hanamura N, Kashikura Y, Takamura M (2012). Role of stromal myofibroblasts in invasive breast cancer: stromal expression of alpha-smooth muscle actin correlates with worse clinical outcome. Breast Cancer.

[26] Im S, Yoo C, Jung JH, Jeon YW, Suh YJ, Lee YS, Choi HJ (2013). Microtubule-associated protein tau, α-tubulin and βIII-tubulin expression in breast cancer. Korean J Pathol.

[27] Li K, Du H, Lian X, Yang S, Chai D, Wang C, Yang R, Chen X (2014). Characterization of β2-microglobulin expression in different types of breast cancer. BMC Cancer.

