# Supplementary material for: A strategy to identify housekeeping genes suitable for analysis in breast cancer diseases
Source: BMC Genomics. 2016 Aug 15;17:639. doi: 10.1186/s12864-016-2946-1 (PMC4986254; doi:10.1186/s12864-016-2946-1)
Supplement: Additional file 1: Table S1. — Primer sequences for gene expression measure by qRT-PCR. (DOC 44 kb) [file 12864_2016_2946_MOESM1_ESM.doc]

**Table S1. Primer sequences for gene expression measure by qRT-PCR.**

| **Primer Name** | **Sequence 5’ – 3’** |
| --- | --- |
| ACTB_F | CACCATTGGCAATGAGCGGTTC |
| ACTB_R | AGGTCTTTGCGGATGTCCACGT |
| GAPDH_F | TGCACCACCAACTGCTTAGC |
| GAPDH_R | GGCATGGACTGTGGTCATGAG |
| B2M_F | TGCTGTCTCCATGTTTGATGTATC |
| B2M_R | TCTCTGCTCCCCACCTCTAAG |
| 18S_F | AACCCGTTGAACCCCATT |
| 18S_R | CCATCCAATCGGTAGTAGCG |
| TUB1A1_F | CGGGCAGTGTTTGTAGACTTGG |
| TUB1A1_R | CTCCTTGCCAATGGTGTAGTGC |
| CCSER2_F | GACAGGAGCATTACCACCTCAG |
| CCSER2_R | CTTCTGAGCCTGGAAAAAGGGC |
| SYMPK_F | CTTCACCAAGGTTGTGCTGGAG |
| SYMPK_R | GCGCTTGAAGATCAGGTCTCGA |
| TMEM11_F | TCCTGGCAGTTTGACCCTTGCT |
| TMEM11_R | CAGTCTCTTTCTGTGCAGGTCG |
| UBXN4_F | GGCAGAAATGGAAGTCAAGAGGG |
| UBXN4_R | GAGGAGCATCAGAAGGGAACTG |
| ANKRD17_F | CAAATGGTGGACACCTCGATGTG |
| ANKRD17_R | CTAAGTAGCGCACCACCTTCAC |
