# Supplementary figures and images for: A strategy to identify housekeeping genes suitable for analysis in breast cancer diseases
Source: BMC Genomics. 2016 Aug 15;17:639. doi: 10.1186/s12864-016-2946-1 (PMC4986254; doi:10.1186/s12864-016-2946-1)

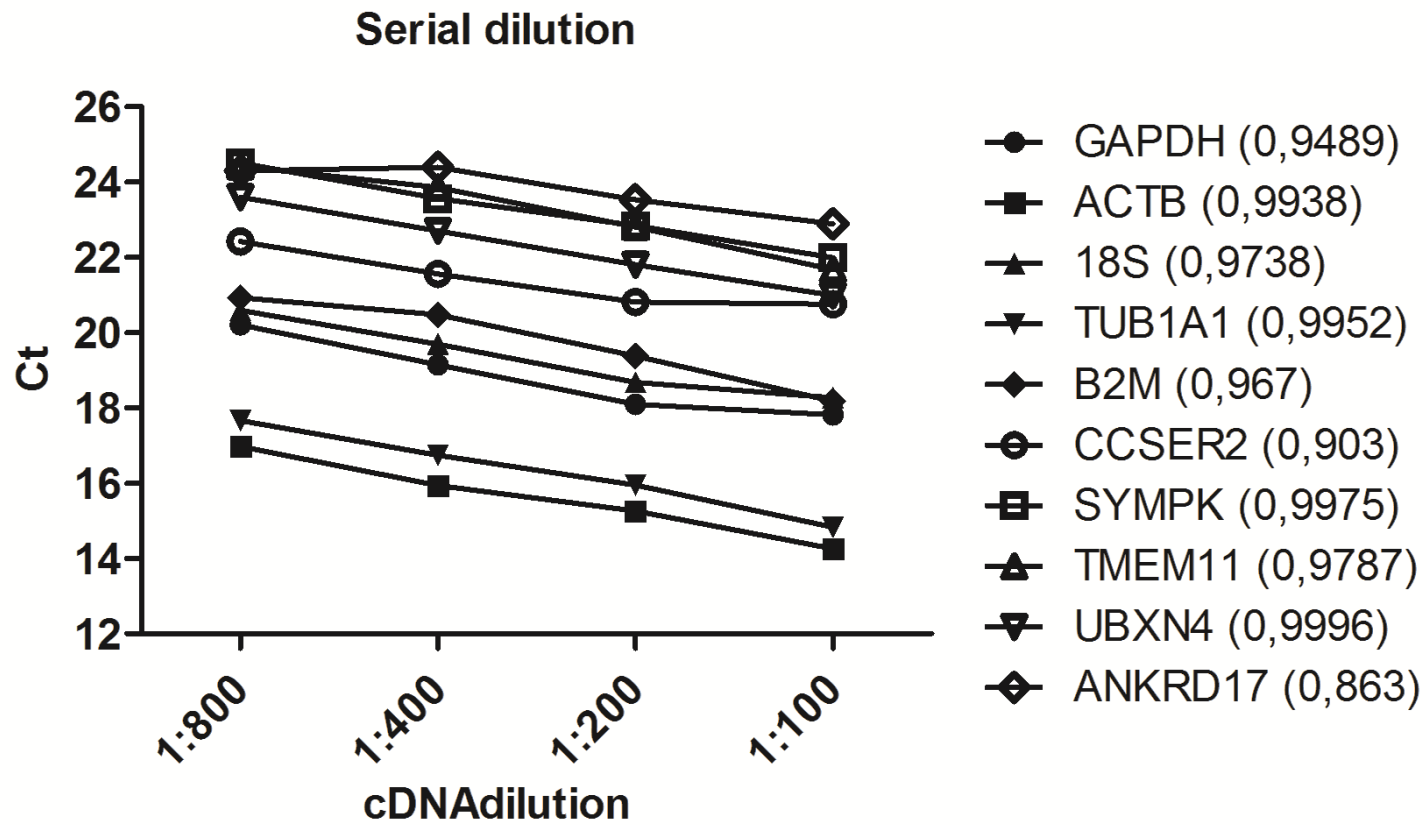

Supplement: Additional file 2: Figure S1. — Standard curve and serial dilutions for nHKGs and tHKGs. The x axis represents the dilution series (1:800, 1:400, 1:200 and 1:100) and the y axis represents the mean of CT for each gene. The correlation coefficient r is given for each gene inside parentheses. (PDF 53 kb) [file 12864_2016_2946_MOESM2_ESM.pdf]

- Activation
- Inhibition
- Binding
- Expression
- Reaction
- Post-transl.
- Catalysis
- Phenotype

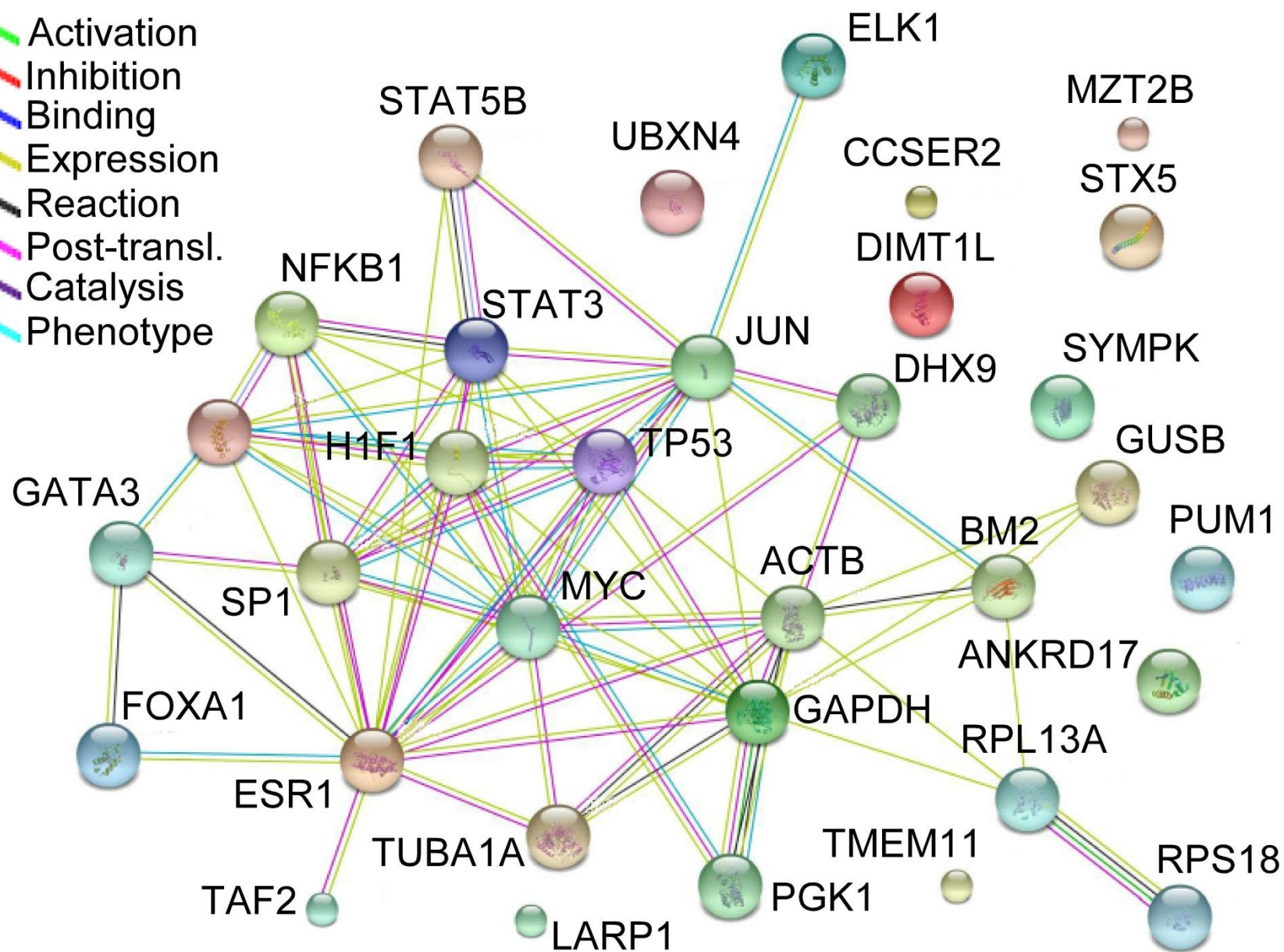

Supplement: Additional file 3: Figure S2. — Subnetworks of nHKGs, tHKGs and transcription factors from STRING in graph layout. Nodes are for genes and links for interaction among them. The color code for edge notation is given on the bottom left. (PDF 301 kb) [file 12864_2016_2946_MOESM3_ESM.pdf]

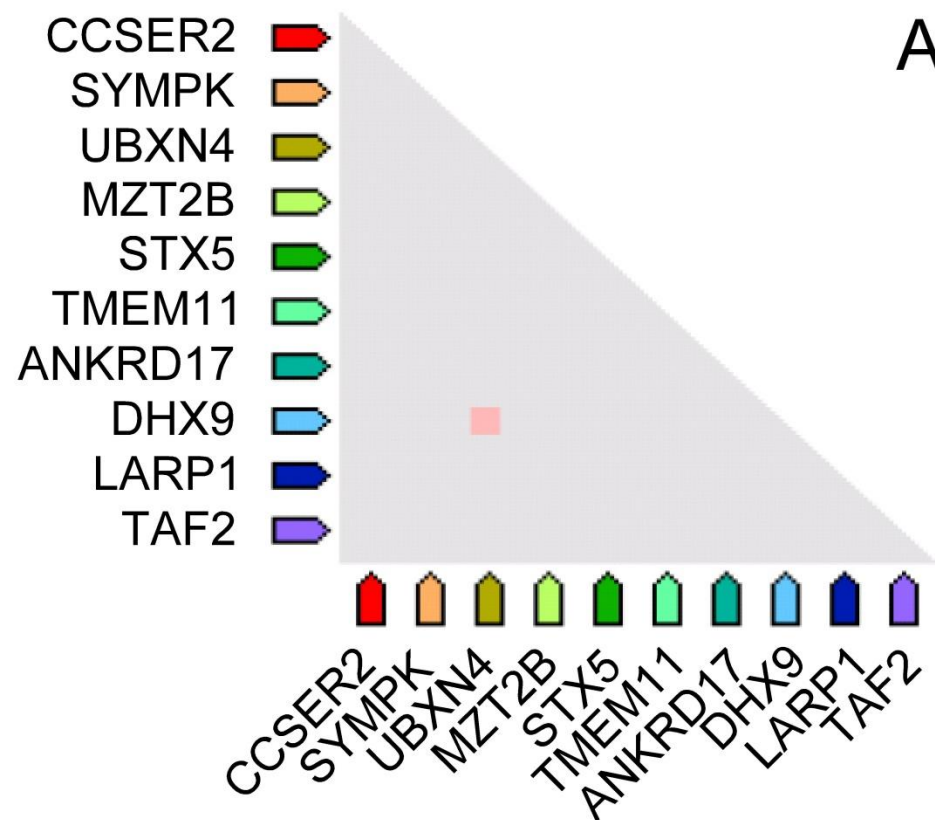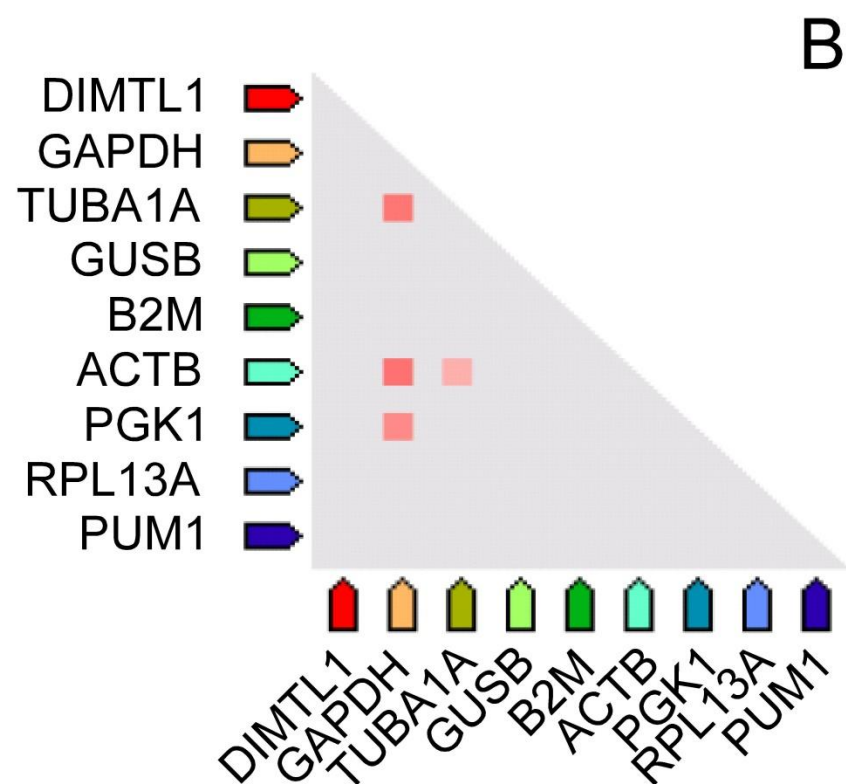

Association score: 0 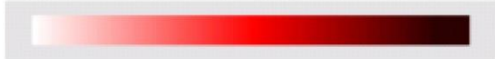 1

Supplement: Additional file 6: Figure S3. — Co-expression of genes in the nHKG and tHKG groups as obtained from STRING. Color intensity represents the association score between each pair of gene nHKGs (A) and tHKGs (B). (PDF 193 kb) [file 12864_2016_2946_MOESM6_ESM.pdf]

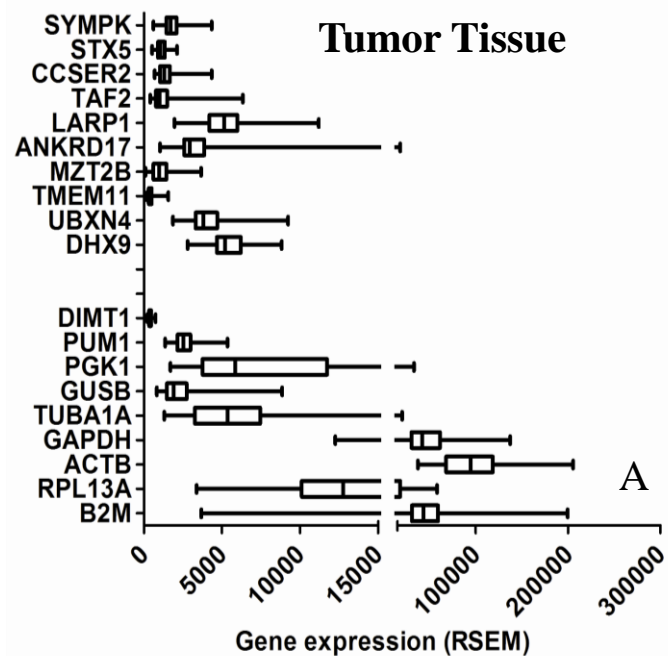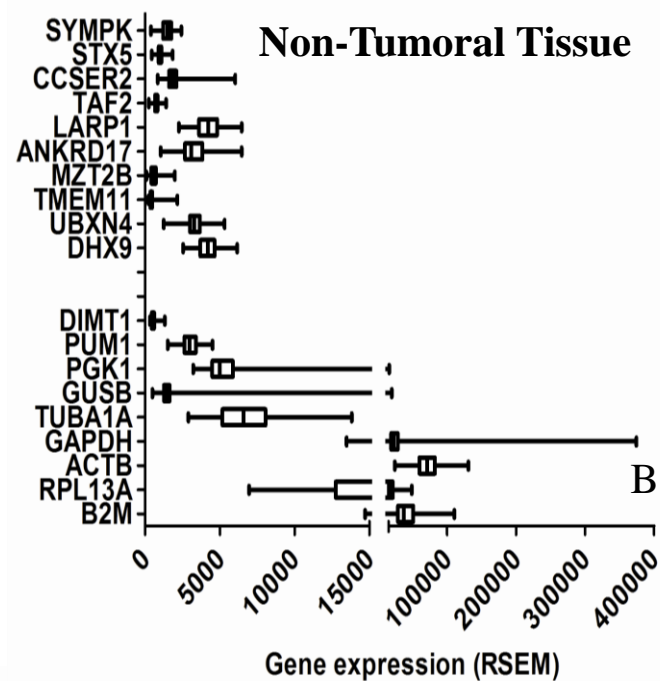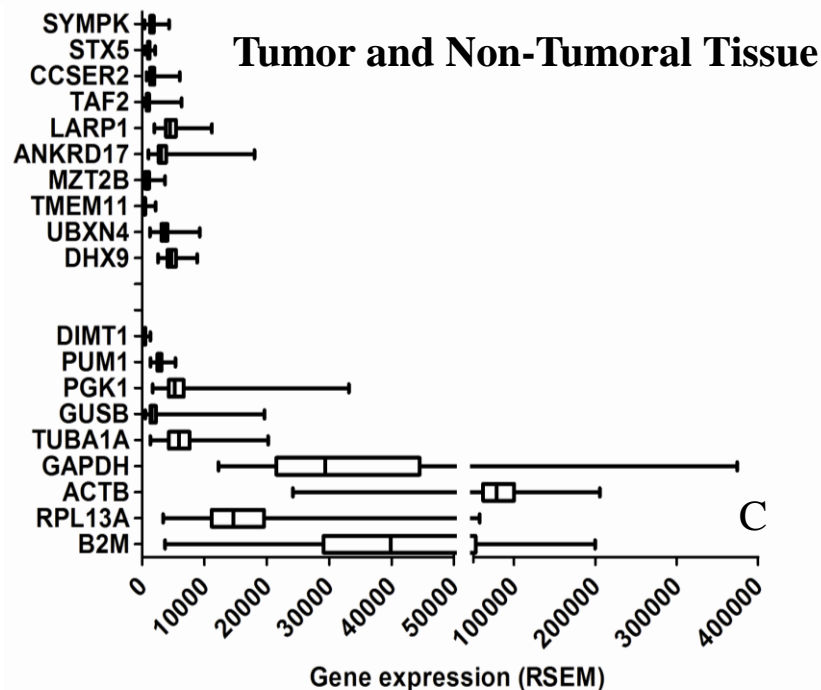

Supplementary Figure 4

Supplement: Additional file 7: Figure S4. — Distribution of expression levels by normalized read counts of 10 nHKGs and 9 tHKGs in breast cancer RNA-seq (n = 95 paired samples) from TCGA. Boxes delimit lower and upper quartiles while vertical lines within boxes indicate median expression values. Lateral whiskers provide lower and higher values as left and right ticks, respectively. (PDF 371 kb) [file 12864_2016_2946_MOESM7_ESM.pdf]

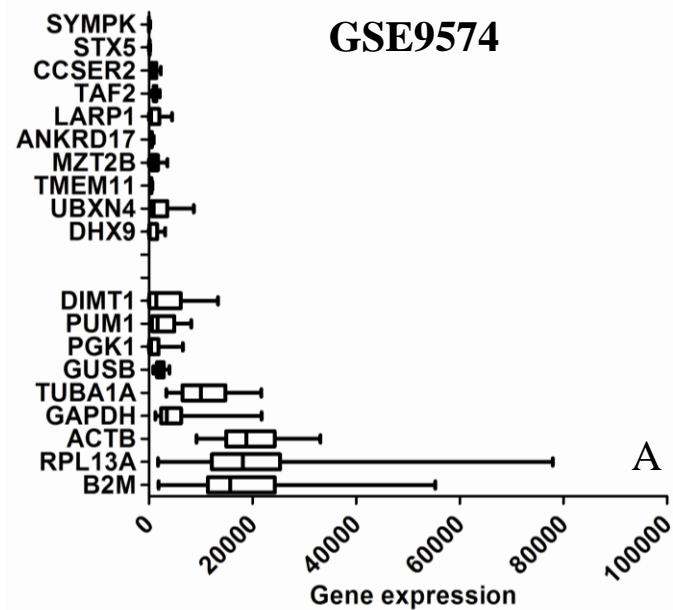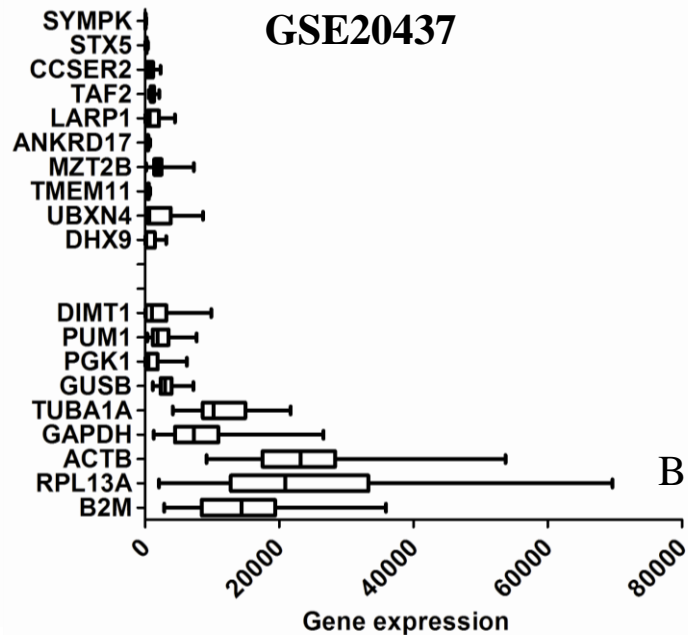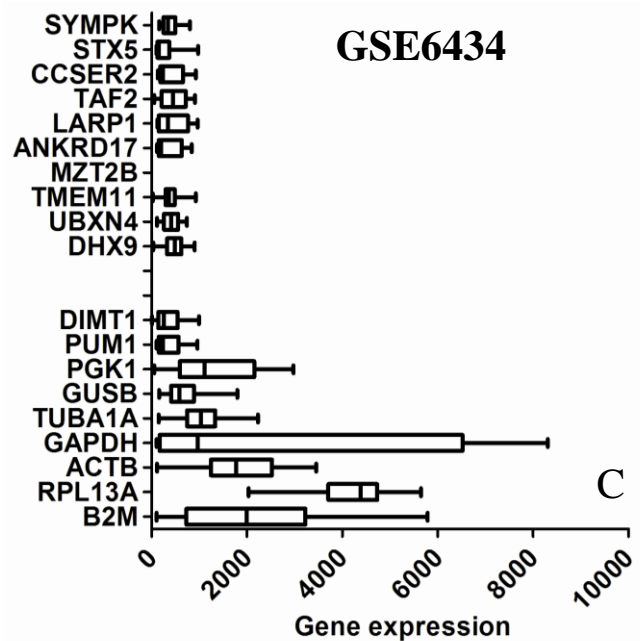

Supplementary Figure 5

Supplement: Additional file 8: Figure S5. — Distribution of expression levels of 10 nHKGs and 9 tHKGs in microarrays of breast cancer samples from the GEO repository. Boxes delimit lower and upper quartiles while the vertical lines within the boxes indicate median expression values. Lateral whiskers provide lower and higher values as left and right ticks, respectively. (PDF 319 kb) [file 12864_2016_2946_MOESM8_ESM.pdf]
