# Supplementary material for: A strategy to identify housekeeping genes suitable for analysis in breast cancer diseases
Source: BMC Genomics. 2016 Aug 15;17:639. doi: 10.1186/s12864-016-2946-1 (PMC4986254; doi:10.1186/s12864-016-2946-1)
Supplement: Additional file 4: Table S2. — Protein-protein interaction for tHKGs and nHKGs. Data from the CCSB interactome database. (DOC 128 kb) [file 12864_2016_2946_MOESM4_ESM.doc]

Table S2. Protein-protein interaction for tHKGs and nHKGs. Data from the CCSB interactome database.

| **Entrez gene IDA** | **Symbol A** | **Entrez gene IDB** | **Symbol B** |
| --- | --- | --- | --- |
| [60](http://www.ncbi.nlm.nih.gov/gene/?term=60) | **ACTB** | [60](http://www.ncbi.nlm.nih.gov/gene/?term=60) | ACTB |
| [60](http://www.ncbi.nlm.nih.gov/gene/?term=60) | **ACTB** | [71](http://www.ncbi.nlm.nih.gov/gene/?term=71) | ACTG1 |
| [60](http://www.ncbi.nlm.nih.gov/gene/?term=60) | **ACTB** | [1072](http://www.ncbi.nlm.nih.gov/gene/?term=1072) | CFL1 |
| [60](http://www.ncbi.nlm.nih.gov/gene/?term=60) | **ACTB** | [1073](http://www.ncbi.nlm.nih.gov/gene/?term=1073) | CFL2 |
| [60](http://www.ncbi.nlm.nih.gov/gene/?term=60) | **ACTB** | [1962](http://www.ncbi.nlm.nih.gov/gene/?term=1962) | EHHADH |
| [60](http://www.ncbi.nlm.nih.gov/gene/?term=60) | **ACTB** | [3192](http://www.ncbi.nlm.nih.gov/gene/?term=3192) | HNRNPU |
| [60](http://www.ncbi.nlm.nih.gov/gene/?term=60) | **ACTB** | [5216](http://www.ncbi.nlm.nih.gov/gene/?term=5216) | PFN1 |
| [60](http://www.ncbi.nlm.nih.gov/gene/?term=60) | **ACTB** | [5340](http://www.ncbi.nlm.nih.gov/gene/?term=5340) | PLG |
| [60](http://www.ncbi.nlm.nih.gov/gene/?term=60) | **ACTB** | [6606](http://www.ncbi.nlm.nih.gov/gene/?term=6606) | SMN1 |
| [60](http://www.ncbi.nlm.nih.gov/gene/?term=60) | **ACTB** | [8507](http://www.ncbi.nlm.nih.gov/gene/?term=8507) | ENC1 |
| [60](http://www.ncbi.nlm.nih.gov/gene/?term=60) | **ACTB** | [10486](http://www.ncbi.nlm.nih.gov/gene/?term=10486) | CAP2 |
| [60](http://www.ncbi.nlm.nih.gov/gene/?term=60) | **ACTB** | [11034](http://www.ncbi.nlm.nih.gov/gene/?term=11034) | DSTN |
| [60](http://www.ncbi.nlm.nih.gov/gene/?term=60) | **ACTB** | [26260](http://www.ncbi.nlm.nih.gov/gene/?term=26260) | FBXO25 |
| [60](http://www.ncbi.nlm.nih.gov/gene/?term=60) | **ACTB** | [55093](http://www.ncbi.nlm.nih.gov/gene/?term=55093) | WDYHV1 |
| [60](http://www.ncbi.nlm.nih.gov/gene/?term=60) | **ACTB** | [653361](http://www.ncbi.nlm.nih.gov/gene/?term=653361) | NCF1 |
| [367](http://www.ncbi.nlm.nih.gov/gene/?term=367) | AR | [2597](http://www.ncbi.nlm.nih.gov/gene/?term=2597) | **GAPDH** |
| [1956](http://www.ncbi.nlm.nih.gov/gene/?term=1956) | EGFR | [2597](http://www.ncbi.nlm.nih.gov/gene/?term=2597) | **GAPDH** |
| [2597](http://www.ncbi.nlm.nih.gov/gene/?term=2597) | **GAPDH** | [2597](http://www.ncbi.nlm.nih.gov/gene/?term=2597) | **GAPDH** |
| [2597](http://www.ncbi.nlm.nih.gov/gene/?term=2597) | **GAPDH** | [4653](http://www.ncbi.nlm.nih.gov/gene/?term=4653) | MYOC |
| [2597](http://www.ncbi.nlm.nih.gov/gene/?term=2597) | **GAPDH** | [5111](http://www.ncbi.nlm.nih.gov/gene/?term=5111) | PCNA |
| [2597](http://www.ncbi.nlm.nih.gov/gene/?term=2597) | **GAPDH** | [5230](http://www.ncbi.nlm.nih.gov/gene/?term=5230) | PGK1 |
| [2597](http://www.ncbi.nlm.nih.gov/gene/?term=2597) | **GAPDH** | [6310](http://www.ncbi.nlm.nih.gov/gene/?term=6310) | ATXN1 |
| [2597](http://www.ncbi.nlm.nih.gov/gene/?term=2597) | **GAPDH** | [6477](http://www.ncbi.nlm.nih.gov/gene/?term=6477) | SIAH1 |
| [87](http://www.ncbi.nlm.nih.gov/gene/?term=87) | ACTN1 | [7846](http://www.ncbi.nlm.nih.gov/gene/?term=7846) | **TUBA1A** |
| [1605](http://www.ncbi.nlm.nih.gov/gene/?term=1605) | DAG1 | [7846](http://www.ncbi.nlm.nih.gov/gene/?term=7846) | **TUBA1A** |
| [3688](http://www.ncbi.nlm.nih.gov/gene/?term=3688) | ITGB1 | [7846](http://www.ncbi.nlm.nih.gov/gene/?term=7846) | **TUBA1A** |
| [7414](http://www.ncbi.nlm.nih.gov/gene/?term=7414) | VCL | [7846](http://www.ncbi.nlm.nih.gov/gene/?term=7846) | **TUBA1A** |
| [7846](http://www.ncbi.nlm.nih.gov/gene/?term=7846) | **TUBA1A** | [90627](http://www.ncbi.nlm.nih.gov/gene/?term=90627) | STARD13 |
| [672](http://www.ncbi.nlm.nih.gov/gene/?term=672) | BRCA1 | [1660](http://www.ncbi.nlm.nih.gov/gene/?term=1660) | **DHX9** |
| [1660](http://www.ncbi.nlm.nih.gov/gene/?term=1660) | **DHX9** | [3609](http://www.ncbi.nlm.nih.gov/gene/?term=3609) | ILF3 |
| [1660](http://www.ncbi.nlm.nih.gov/gene/?term=1660) | **DHX9** | [5970](http://www.ncbi.nlm.nih.gov/gene/?term=5970) | RELA |
| [1660](http://www.ncbi.nlm.nih.gov/gene/?term=1660) | **DHX9** | [10657](http://www.ncbi.nlm.nih.gov/gene/?term=10657) | KHDRBS1 |
| [1660](http://www.ncbi.nlm.nih.gov/gene/?term=1660) | **DHX9** | [26993](http://www.ncbi.nlm.nih.gov/gene/?term=26993) | AKAP8L |
| [3297](http://www.ncbi.nlm.nih.gov/gene/?term=3297) | HSF1 | [8189](http://www.ncbi.nlm.nih.gov/gene/?term=8189) | **SYMPK** |
| [8189](http://www.ncbi.nlm.nih.gov/gene/?term=8189) | **SYMPK** | [29101](http://www.ncbi.nlm.nih.gov/gene/?term=29101) | SSU72 |
| [664](http://www.ncbi.nlm.nih.gov/gene/?term=664) | BNIP3 | [8834](http://www.ncbi.nlm.nih.gov/gene/?term=8834) | **TMEM11** |
| [665](http://www.ncbi.nlm.nih.gov/gene/?term=665) | BNIP3L | [8834](http://www.ncbi.nlm.nih.gov/gene/?term=8834) | **TMEM11** |
| [8834](http://www.ncbi.nlm.nih.gov/gene/?term=8834) | **TMEM11** | [89885](http://www.ncbi.nlm.nih.gov/gene/?term=89885) | **FATE1** |
| [8834](http://www.ncbi.nlm.nih.gov/gene/?term=8834) | **TMEM11** | [90993](http://www.ncbi.nlm.nih.gov/gene/?term=90993) | CREB3L1 |
| [23190](http://www.ncbi.nlm.nih.gov/gene/?term=23190) | **UBXN4** | [29979](http://www.ncbi.nlm.nih.gov/gene/?term=29979) | UBQLN1 |
| [23367](http://www.ncbi.nlm.nih.gov/gene/?term=23367) | **LARP1** | [147872](http://www.ncbi.nlm.nih.gov/gene/?term=147872) | CCDC155 |
| [4105](http://www.ncbi.nlm.nih.gov/gene/?term=4105) | MAGEA6 | [6811](http://www.ncbi.nlm.nih.gov/gene/?term=6811) | **STX5** |
| [6810](http://www.ncbi.nlm.nih.gov/gene/?term=6810) | STX4 | [6811](http://www.ncbi.nlm.nih.gov/gene/?term=6811) | **STX5** |
| [6811](http://www.ncbi.nlm.nih.gov/gene/?term=6811) | **STX5** | [6867](http://www.ncbi.nlm.nih.gov/gene/?term=6867) | TACC1 |
| [6811](http://www.ncbi.nlm.nih.gov/gene/?term=6811) | **STX5** | [8775](http://www.ncbi.nlm.nih.gov/gene/?term=8775) | NAPA |
| [6811](http://www.ncbi.nlm.nih.gov/gene/?term=6811) | **STX5** | [9527](http://www.ncbi.nlm.nih.gov/gene/?term=9527) | GOSR1 |
| [6811](http://www.ncbi.nlm.nih.gov/gene/?term=6811) | **STX5** | [9570](http://www.ncbi.nlm.nih.gov/gene/?term=9570) | GOSR2 |
| [6811](http://www.ncbi.nlm.nih.gov/gene/?term=6811) | **STX5** | [63908](http://www.ncbi.nlm.nih.gov/gene/?term=63908) | NAPB |
| [6811](http://www.ncbi.nlm.nih.gov/gene/?term=6811) | **STX5** | [147872](http://www.ncbi.nlm.nih.gov/gene/?term=147872) | CCDC155 |
