# Supplementary material for: A strategy to identify housekeeping genes suitable for analysis in breast cancer diseases
Source: BMC Genomics. 2016 Aug 15;17:639. doi: 10.1186/s12864-016-2946-1 (PMC4986254; doi:10.1186/s12864-016-2946-1)
Supplement: Additional file 5: Table S3. — Protein-protein interaction for tHKGs and nHKGs. Data from the cancer-systemsbiology interactome database. (DOC 232 kb) [file 12864_2016_2946_MOESM5_ESM.doc]

Table S3. Protein-protein interaction for tHKGs and nHKGs. Data from the cancer-systemsbiology interactome database.

| **Symbol A** | **Symbol B** | **Interaction type** |
| --- | --- | --- |
| **GAPDH** | CREBBP | Pos^1^ |
| CAMK1 | **GAPDH** | Pos |
| MAX | **GAPDH** | Pos |
| MYC | **GAPDH** | Pos |
| PTK2B | **GAPDH** | Pos |
| RAB2A | **GAPDH** | Pos |
| DLD | **GAPDH** | Phy^2^ |
| **GAPDH** | CSF1 | Pos |
| ESR1 | **GAPDH** | Pos |
| **GAPDH** | HSPB1 | Phy |
| **GAPDH** | AR | Pos |
| SCYL2 | **GAPDH** | Pos |
| CDK4 | **GAPDH** | Pos |
| **GAPDH** | MAOB | Pos |
| FLNA | **ACTB** | Phy |
| **ACTB** | GTF2F1 | Pos |
| PRKCB | **ACTB** | Pos |
| MYL12B | **ACTB** | Pos |
| PFN3 | **ACTB** | Phy |
| TJP3 | **ACTB** | Phy |
| **ACTB** | GTF2A1 | Pos |
| **ACTB** | PLD1 | Neg^3^ |
| RHOA | **ACTB** | Pos |
| PFN1 | **ACTB** | Phy |
| CFL2 | **ACTB** | Phy |
| WASF3 | **ACTB** | Pos |
| CTTN | **ACTB** | Phy |
| **ACTB** | ACTN2 | Phy |
| **ACTB** | TJP1 | Phy |
| **ACTB** | EZR | Phy |
| **ACTB** | LOC653888 | Phy |
| PIP5K1C | **ACTB** | Phy |
| **ACTB** | PARVG | Phy |
| **ACTB** | POLR2A | Pos |
| ARPC1B | **ACTB** | Phy |
| CFL1 | **ACTB** | Phy |
| ARPC1A | **ACTB** | Phy |
| **ACTB** | ARPC5L | Phy |
| **ACTB** | GTF2B | Pos |
| **ACTB** | TLN1 | Phy |
| **ACTB** | TJP2 | Phy |
| RDX | **ACTB** | Phy |
| ARPC5 | **ACTB** | Phy |
| WAS | **ACTB** | Pos |
| PAK1 | **ACTB** | Phy |
| **ACTB** | VASP | Phy |
| MYH11 | **ACTB** | Phy |
| **ACTB** | TMSL3 | Phy |
| PRKCD | **ACTB** | Pos |
| **ACTB** | TMSB4Y | Phy |
| **ACTB** | ABL1 | Neg |
| WASF2 | **ACTB** | Pos |
| PARVA | **ACTB** | Phy |
| PFN2 | **ACTB** | Phy |
| MYL12A | **ACTB** | Pos |
| **ACTB** | VCL | Phy |
| **ACTB** | TAF1 | Pos |
| **ACTB** | NEXN | Phy |
| HSPB1 | **ACTB** | Phy |
| CTNNA2 | **ACTB** | Phy |
| MYH9 | **ACTB** | Phy |
| **ACTB** | CFL1 | Neg |
| MYH10 | **ACTB** | Phy |
| MYL5 | **ACTB** | Pos |
| PRKCE | **ACTB** | Pos |
| **ACTB** | KEAP1 | Phy |
| PRKD1 | **ACTB** | Phy |
| CSRP1 | **ACTB** | Phy |
| WASF1 | **ACTB** | Pos |
| **ACTB** | ARHGAP24 | Phy |
| CNN1 | **ACTB** | Pos |
| PRKCG | **ACTB** | Pos |
| MYH8 | **ACTB** | Phy |
| CTNNA1 | **ACTB** | Phy |
| **ACTB** | NOS3 | Pos |
| MYL10 | **ACTB** | Pos |
| **ACTB** | ACTN3 | Phy |
| **ACTB** | GTF3A | Pos |
| PRKCQ | **ACTB** | Pos |
| **ACTB** | ACTN4 | Phy |
| ARPC3 | **ACTB** | Phy |
| MYH6 | **ACTB** | Phy |
| ACTB | NR3C1 | Pos |
| PFN4 | **ACTB** | Phy |
| MYH15 | **ACTB** | Phy |
| **ACTB** | TMSB4X | Phy |
| MYH4 | **ACTB** | Phy |
| **ACTB** | ZYX | Phy |
| MYH3 | **ACTB** | Phy |
| CTNNA3 | **ACTB** | Phy |
| **ACTB** | GTF2E1 | Pos |
| PRKCA | **ACTB** | Pos |
| **ACTB** | TNS1 | Phy |
| MSN | **ACTB** | Phy |
| MARCKS | **ACTB** | Pos |
| LASP1 | **ACTB** | Phy |
| ARPC4 | **ACTB** | Phy |
| **ACTB** | ACTN1 | Phy |
| WASL | **ACTB** | Pos |
| BAIAP2 | **ACTB** | Pos |
| MYL7 | **ACTB** | Pos |
| MLLT4 | **ACTB** | Pos |
| PRKCH | **ACTB** | Pos |
| MYL9 | **ACTB** | Pos |
| MYLPF | **ACTB** | Pos |
| PLEC | **ACTB** | Phy |
| IQGAP1 | **ACTB** | Pos |
| PARVB | **ACTB** | Phy |
| ACTB | MYH14 | Phy |
| MYH7B | **ACTB** | Phy |
| ARPC2 | **ACTB** | Phy |
| **ACTB** | MYL10 | Phy |
| MYH2 | **ACTB** | Phy |
| **ACTB** | IFNB1 | Pos |
| **ACTB** | MYH13 | Phy |
| MYL2 | **ACTB** | Pos |
| MYH1 | **ACTB** | Phy |
| **TUBA1A** | CEP250 | Phy |
| **TUBA1A** | OFD1 | Phy |
| **TUBA1A** | TUBB1 | Phy |
| **TUBA1A** | CP110 | Phy |
| **TUBA1A** | CEP70 | Phy |
| **TUBA1A** | DYNC1H1 | Phy |
| **TUBA1A** | CEP164 | Phy |
| **TUBA1A** | HAUS2 | Phy |
| **TUBA1A** | CETN2 | Phy |
| **TUBA1A** | NEDD1 | Phy |
| **TUBA1A** | PRKAR2B | Phy |
| **TUBA1A** | TUBB4 | Phy |
| **TUBA1A** | CBL | Phy |
| **TUBA1A** | CEP135 | Phy |
| **TUBA1A** | AZI1 | Phy |
| **TUBA1A** | CKAP5 | Phy |
| **TUBA1A** | SFI1 | Phy |
| **TUBA1A** | SSNA1 | Phy |
| **TUBA1A** | CEP290 | Phy |
| **TUBA1A** | MAPRE1 | Phy |
| ACTR1A | **TUBA1A** | Phy |
| AKAP9 | **TUBA1A** | Phy |
| ALMS1 | **TUBA1A** | Phy |
| CDK1 | **TUBA1A** | Phy |
| CDK5RAP2 | **TUBA1A** | Phy |
| CENPJ | **TUBA1A** | Phy |
| CEP110 | **TUBA1A** | Phy |
| CEP152 | **TUBA1A** | Phy |
| CEP57 | **TUBA1A** | Phy |
| CEP63 | **TUBA1A** | Phy |
| CEP72 | **TUBA1A** | Phy |
| CEP76 | **TUBA1A** | Phy |
| CEP78 | **TUBA1A** | Phy |
| CLASP1 | **TUBA1A** | Phy |
| CSNK1D | **TUBA1A** | Phy |
| CSNK1E | **TUBA1A** | Phy |
| DCTN1 | **TUBA1A** | Phy |
| DCTN2 | **TUBA1A** | Phy |
| DCTN3 | **TUBA1A** | Phy |
| DNM2 | **TUBA1A** | Phy |
| DYNC1I2 | **TUBA1A** | Phy |
| DYNLL1 | **TUBA1A** | Phy |
| ESR1 | **TUBA1A** | Pos |
| FGFR1OP | **TUBA1A** | Phy |
| FYN | **TUBA1A** | Phy |
| GJA1 | **TUBA1A** | Phy |
| GRB2 | **TUBA1A** | Phy |
| HSP90AA1 | **TUBA1A** | Phy |
| NDE1 | **TUBA1A** | Phy |
| NEK2 | **TUBA1A** | Phy |
| NINL | **TUBA1A** | Phy |
| ODF2 | **TUBA1A** | Phy |
| PAFAH1B1 | **TUBA1A** | Phy |
| PCM1 | **TUBA1A** | Phy |
| PCNT | **TUBA1A** | Phy |
| PIK3CA | **TUBA1A** | Phy |
| PLK1 | **TUBA1A** | Phy |
| PLK4 | **TUBA1A** | Phy |
| PPP2R1A | **TUBA1A** | Phy |
| PRKACA | **TUBA1A** | Phy |
| PXN | **TUBA1A** | Phy |
| SDCCAG8 | **TUBA1A** | Phy |
| SYK | **TUBA1A** | Pos |
| TP73 | **TUBA1A** | Pos |
| TSGA14 | **TUBA1A** | Phy |
| TUBA4A | **TUBA1A** | Phy |
| TUBB | **TUBA1A** | Phy |
| TUBB2C | **TUBA1A** | Phy |
| TUBG1 | **TUBA1A** | Phy |
| YWHAE | **TUBA1A** | Phy |
| YWHAG | **TUBA1A** | Phy |
| CD247 | **B2M** | Phy |
| CD28 | **B2M** | Neg |
| CD3D | **B2M** | Phy |
| CD3G | **B2M** | Phy |
| CD80 | **B2M** | Neg |
| CD86 | **B2M** | Neg |
| CD8A | **B2M** | Phy |
| CD8B | **B2M** | Phy |
| HLA-A | **B2M** | Phy |
| IFNA1 | **B2M** | Pos |
| IFNG | **B2M** | Pos |
| LCK | **B2M** | Phy |
| TRA | **B2M** | Phy |
| TRB | **B2M** | Phy |
| **B2M** | AR | Neg |
| **B2M** | CD247 | Phy |
| **B2M** | CD28 | Neg |
| **B2M** | CD3D | Phy |
| **B2M** | CD3E | Phy |
| **B2M** | CD3G | Phy |
| **B2M** | CD80 | Neg |
| **B2M** | CD86 | Neg |
| **B2M** | CD8A | Phy |
| **B2M** | CD8B | Phy |
| **B2M** | CSK | Neg |
| **B2M** | EOMES | Pos |
| **B2M** | GRAP2 | Pos |
| **B2M** | GZMB | Pos |
| **B2M** | HLA-E | Pos |
| **B2M** | IL2 | Pos |
| **B2M** | IL2RA | Pos |
| **B2M** | IL2RB | Pos |
| **B2M** | IL2RG | Pos |
| **B2M** | JUN | Pos |
| **B2M** | LAT | Pos |
| **B2M** | LCP2 | Pos |
| **B2M** | PAG1 | Neg |
| **B2M** | PIK3R1 | Pos |
| **B2M** | PRF1 | Pos |
| **B2M** | PRKCQ | Pos |
| **B2M** | PTPN6 | Pos |
| **B2M** | SHC1 | Pos |
| **B2M** | SHC2 | Pos |
| **B2M** | SHC3 | Pos |
| **B2M** | TRA | Phy |
| **B2M** | TRB | Phy |
| **B2M** | VAV1 | Pos |
| **B2M** | ZAP70 | Phy |
| CDK6 | **PUM1** | Pos |
| PIM2 | **PUM1** | Pos |
| ARNT | **PGK1** | Pos |
| EPAS1 | **PGK1** | Pos |
| HIF1A | **PGK1** | Pos |
| **PGK1** | PFKFB1 | Pos |
| PAK4 | **LARP1** | Pos |
| PRKACA | **LARP1** | Pos |
| GSK3A | **LARP1** | Pos |
| PRKCZ | **LARP1** | Pos |
| **DHX9** | IL4 | Pos |
| **DHX9** | NFKB1 | Pos |
| **DHX9** | ABCB1 | Neg |

^1^Pos: positive (activation); ^2^Phy: physical; ^3^Neg: negative (inhibition).
